# Chest Wall Reconstruction With Strattice in an Immunosuppressed Patient

**Published:** 2011-11-23

**Authors:** Karen M. Kaplan, Karan Chopra, Jeffrey Feiner, Brian R. Gastman

**Affiliations:** ^a^Temple University School of Medicine, Philadelphia, PA; ^b^University of Maryland School of Medicine, Baltimore; ^c^Johns Hopkins Hospital, Baltimore, MD; ^d^Cleveland Clinic, Cleveland, OH

## Abstract

We report successful reconstruction of a challenging composite chest wall defect in an immunocompromised patient using a biologic mesh. Infection results in significant morbidity and mortality in immunocompromised patients. Thus, reconstruction in this population requires careful selection of appropriate materials to repair the defect. A 26-year-old woman with a cardiac paraganglioma required resection of the heart, portions of the great vessels, several ribs, and a large portion of the sternum, with subsequent orthotopic cardiac transplantation. Titanium plates were used to restore sternal continuity and Strattice was used for chest wall reconstruction. Strattice was selected due to its ability to become incorporated and resist wound infection, to provide stability to the rib cage, and to protect the newly transplanted heart. In our experience, Strattice provides a viable alternative to other biologics and is a safer alternative to synthetic mesh for chest wall reconstruction in immunocompromised patients.

Each year, 3500 orthotopic heart transplants are performed worldwide, of which a small percentage are for primary cardiac tumors.^1^ Treatment of these tumors may require extensive resection of the chest wall, in addition to a heart transplant, resulting in composite chest wall defects that require reconstruction.

Cardiac transplant patients are maintained on lifelong immunosuppression, which can influence the method of chest wall reconstruction. Chest wall skeletal defects have traditionally been repaired using Prolene or polytetraflouroethylene mesh, Gore-Tex soft-tissue patch, or a methyl methacrylate sandwich.^2^ Unfortunately, these synthetic materials are unable to resist infection, which is a devastating complication in heart transplant patients, accounting for 30% of the mortality in the first year.^1^^-^3 The risk of infection in an immunocompetent patient is 0.4% to 5.1% following median sternotomy; however, in immunosuppressed patients, the risk is much higher.^4^^-^6 These patients can benefit significantly from chest wall reconstruction using a biologic mesh that promotes tissue incorporation and resists infection.

We report the use of non–cross-linked acellular porcine dermal matrix (Strattice, Lifecell Corporation, Branchburg, New Jersey) to reconstruct a large, composite chest wall defect in an orthotopic heart transplant patient following resection of a primary cardiac paraganglioma. The use of Strattice for chest wall reconstruction in an immunosuppressed patient has not been reported previously.

## CASE

**Figure 1:**
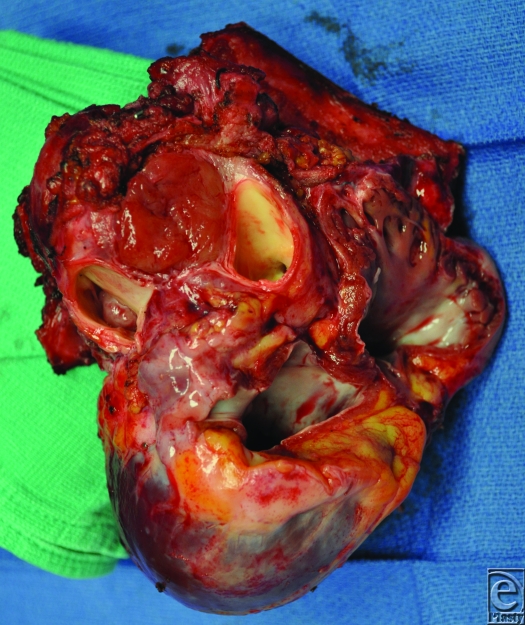
The intraoperative specimen of heart, great vessels, and adjacent structures.

**Figure 2:**
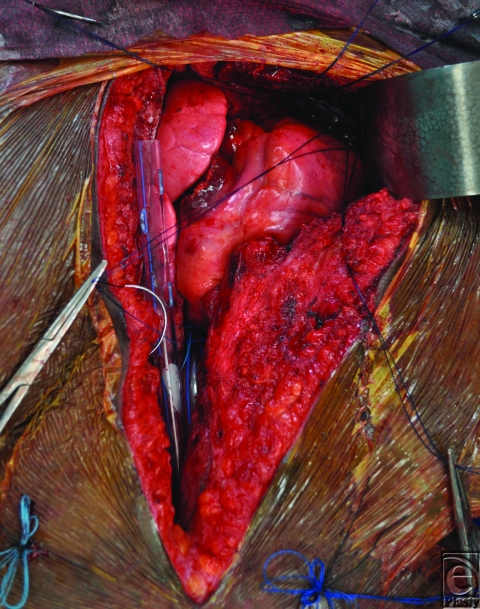
A 10 × 10 cm^2^ chest wall defect after en bloc resection of specimen.

**Figure 3:**
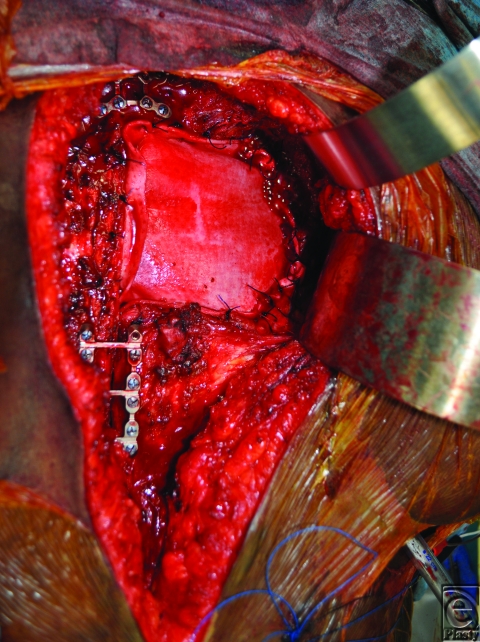
Intraoperative photos showing the biologic mesh secured in place to replace the missing segment of chest wall.

A 26-year-old African American woman presented for an orthotopic heart transplantation following staged resection for a cardiac paraganglioma. The oncologic treatment included an en bloc resection of the heart, portions of the great vessels, several ribs and a portion of pectoralis major muscle (Fig 1). The plastic surgery department evaluated the patient intraoperatively for chest wall reconstruction. It was noted that the sternum was divided in the midline and a large portion of the left sternal body, along with 4 ribs to the anterior axillary line, had been resected en bloc with the specimen. This resulted in a 10 × 10 cm^2^ chest wall defect (Fig 2), and a decision was made to proceed with Strattice for chest wall reconstruction. Multiple interrupted 0 polydioxanone suture (PDS) sutures were placed in horizontal mattress fashion 270° around the defect through the ribs and intercostal musculature. The Strattice was trimmed to fit the chest wall defect and was parachuted underneath the rib edges to minimize contact between the bony edges and the heart. The Strattice was then serially secured in place, creating a tight construct to replace the missing segment of chest wall (Fig 3). Bone reduction clamps were used to reduce and align the manubrium and residual body of the sternum. Rigid fixation was provided with a locking sternal plating system. The medial edge of the Strattice was then aligned with the contralateral sternum/ribs and this was secured with interrupted figure of eight 0 PDS sutures.

**Figure 4:**
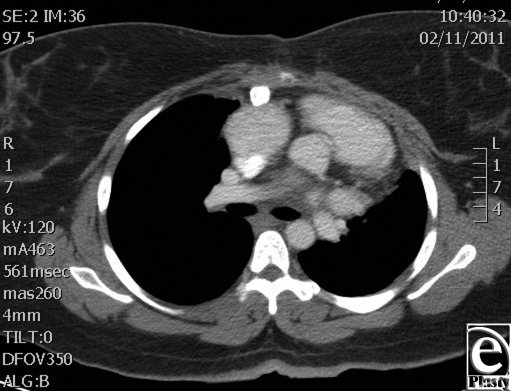
Computed tomographic image shows stable internal reconstruction at 9 months with no fluid collection around the graft.

**Figure 5:**
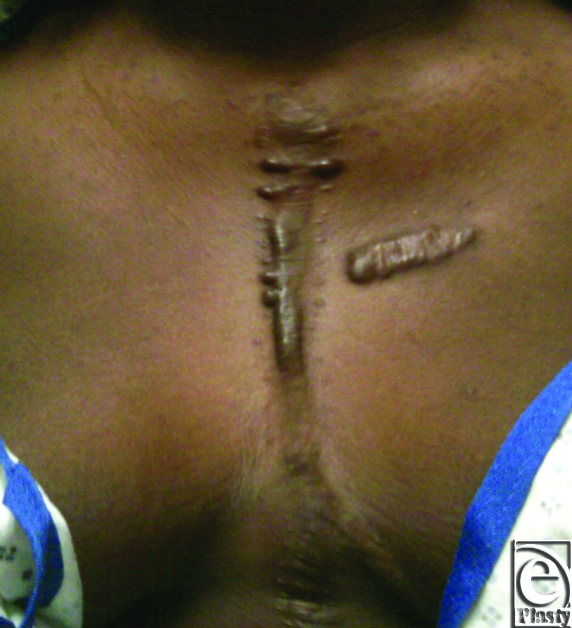
At 14-month follow-up, the scar is well healed with no signs of infection or dehiscence. A prior keloid on breast and mild keloid from sternotomy incision are present.

Muscle coverage for the implants was obtained by elevating the residual pectoralis muscle on the left and approximating this to a composite pectoralis myocutaneous flap that was elevated from the right chest. The patient did well postoperatively and was discharged home on postoperative day 16. A 9-month computed tomographic scan shows stable internal reconstruction and no fluid collection around the graft (Fig 4). At a 14-month postoperative visit, her chest wall construct was stable and healing without evidence of infection. A 14-month follow-up image shows the scar is well healed and without signs of infection or dehiscence (Fig 5).

## DISCUSSION

Infection results in significant morbidity and mortality in immunocompromised patients. Reconstruction in this population requires selection of appropriate materials to repair the defect. Synthetic mesh is susceptible to infection and vascularized tissue flaps may provide coverage but lack structural support for larger defects. Therefore, chest wall reconstruction with biologic materials is a safer alternative that will resist infection and provide structural support.^7^

AlloDerm (Lifecell Corporation, Branchburg, New Jersey) human acellular dermal matrix is a biologic mesh that has been studied extensively in humans and animals. The AlloDerm tissue matrix is integrated into the body through rapid revascularization, white cell migration, and cell repopulation, which allows it to resist infection and allows for local treatment of infection.^7^ Cothren et al^8^ reported a case in which AlloDerm was used in combination with a latissimus dorsi muscle flap to reconstruct a 20 × 20 cm^2^ chest wall defect after resection of a spindle cell sarcoma. They found it to be an ideal material, providing adequate structural stability with a reduction in the incidence of infection. One of its drawbacks for chest wall reconstruction, however, is its tendency to stretch over time.^9^

Strattice is a biologic mesh made from porcine dermal matrix that incorporates much like AlloDerm. Strattice has been shown in primate models to become revascularized in the abdominal wall without immunologic rejection.^10^ The use of Strattice has also been reported as reinforcement for component separation in ventral hernia repair where it has also been found to adequately resist infection.^11^

Despite the ability of both AlloDerm and Strattice to become revascularized and resist infection, there are some important differences that make Strattice a preferred biologic mesh for this indication. Strattice is less elastic and can be harvested in larger and more consistent sheets, allowing for repair of larger defects with a single piece. In addition, the supply of porcine skin is greater than that of human cadaveric skin and can be easily obtained at a substantially reduced cost.^12^^,^^13^ Perhaps its greatest feature is its ability to resist stretching, while still being incorporated like its allogenic counterparts.^12^ In short, these qualities make Strattice an ideal biologic mesh for the repair of large chest wall defects in immunosuppressed patients.

In this case, we used titanium plates to rigidly fixate the remaining sternum and manubrium. Rigid fixation is associated with improved bone healing and decreased bone infection.^4^ If, however, there was an early wound infection, the titanium plates could be removed while leaving the Strattice in place, protecting the heart. In the later phases of convalescence, the hardware could also be removed (after bony union) while still maintaining durable cardiac coverage with Strattice.
